# The Evaluation of the Effectiveness of Adsorbents Based on Acrylamide Hydrogels and Cryogels for Water Purification from Radioactive Contaminants

**DOI:** 10.3390/gels11050311

**Published:** 2025-04-22

**Authors:** Yuriy Artamonov, Pavel Krivitskiy, Fail Zhamaldinov, Vladimir Aseyev, Alexey Klivenko

**Affiliations:** 1 Institute of Radiation Safety and Ecology of National Nuclear Center of the Republic of Kazakhstan, Kurchatov 180010, Kazakhstan; krivitskiy@nnc.kz (P.K.); zhamaldinov@nnc.kz (F.Z.); 2 Department of Chemistry, University of Helsinki, 00014 Helsinki, Finland; vladimir.aseyev@helsinki.fi; 3 Department of Chemistry and Ecology, Research School of Physical and Chemical Sciences, Shakarim University of Semey, Semey 071412, Kazakhstan; alexeyklivenko@mail.ru

**Keywords:** hydrogel, cryogel, acrylamide, selective adsorption, polymer gels, ^137^Cs, ^90^Sr, activity concentration

## Abstract

The article presents the assessment results on the effectiveness of polymer hydrogel and cryogel-based adsorbents for treating natural waters from radioactive contaminants. Nine polymer gels were synthesized, their physico-chemical properties studied, and the processes of selective adsorption of radioactive elements such as ^137^Cs, ^90^Sr, and other chemical elements in aqueous solutions were investigated using polymer gels. The effectiveness of radioactive element adsorption from aqueous solutions by polymer hydrogels and cryogels was evaluated by placing different pure samples of the synthesized gels in radioactively contaminated aqueous solutions. At the same time, the activity of the aqueous solution was measured before placing the polymer gel into it. Then, the polymer gel was placed in the aqueous solution for 3 days. Afterward, the activity of the polymer gel was measured after it was extracted from the aqueous solution. The best adsorption characteristics and selectivity with respect to ^137^Cs was demonstrated by hydrogels AM:AA/3—2.4 × 10^−8^ mg/g, AM:AA/2—4.1 × 10^−9^ mg/g, and AM:AA/5—3.7 × 10^−9^ mg/g. Cryogel MAA:AM—7.0 × 10^−8^ mg/g, hydrogel AM:AA/2—5.1 × 10^−8^ mg/g, and hydrogel AM:AA/5—1.5 × 10^−8^ mg/g exhibit the highest selectivity for ^90^Sr. An adsorption potential has been demonstrated by the synthesized polymer gels with respect to such chemical elements as K, Fe, Ni, and U.

## 1. Introduction

Nuclear power plants, facilities for the production of nuclear fuel cycle materials, radioactively contaminated areas of nuclear test sites, as well as sites for the disposal of radioactive industrial waste pose a threat to the contamination of aquatic ecosystems. At the same time, the aquatic environment is one of the main migration pathways of radionuclides far beyond the industrial sites, test locations, and tailings dumps [1]. Together with the adsorption processes of radionuclide deposition in bottom sediments [2,3], they are transported and accumulated by the food chain [4], further involved in the biotic cycle as a result of the vital activities of living organisms [5]. Dilution of radioactive elements in the aquatic environment is important, which leads to the spread of contamination and a worse radioecological situation in vast spaces. The situation is aggravated by the vastness of the hydrological network of radionuclide migration, in which sources of domestic water supply may be a final critical link.

^137^Cs and ^90^Sr are the most hazardous radionuclides due to the emission of gamma radiation and high-energy beta particles, respectively, as well as the high probability of their formation during the nuclear fission process. They cause long-term damage to ecosystems due to their long half-life, approximately 30 years, and high mobility in the aquatic environment. However, the removal of these radionuclides from ecological systems is quite challenging, as the radionuclides are present in very low concentrations and coexist with a large excess of competing cations (Na^+^, K^+^, Mg^2+^, Ca^2+^, and others) [6,7].

Currently, common water purification methods include chemical precipitation, coagulation, membrane filtration, electrodialysis, ion exchange, and adsorption, with adsorption being the most widely used due to its high removal efficiency, simplicity, and environmental friendliness. In the adsorption process, the adsorbent is key to achieving effective removal of pollutants [8,9,10,11,12]. Therefore, the use of polymer gels as adsorbents for contaminant elements has been a focus of researchers for a long time [13,14]. The interest of scientists is driven by the potential applications of such gels in medicine, nanotechnology, catalysis, and ecology. Due to their porous structure, excellent water adsorption capacity, simplicity, and low synthesis cost, hydrogels are widely used for the removal of pollutants.

In publications, polymer hydrogels [15,16,17,18,19,20,21] and cryogels [22,23,24,25,26,27,28,29,30] are considered as adsorbents for dyes [31,32,33,34], metal ions [35,36,37,38], oil pollutants [39,40], and radionuclides [41,42,43]. There is a lack of information in the field of gel materials created for the adsorption of radionuclides in aqueous solutions. Various adsorbents have been studied for the removal of ^137^Cs and ^90^Sr from water; however, these adsorbents are either expensive or lack the necessary efficiency for large-scale water purification due to their low selectivity for Cs and Sr.

This study is the first to investigate the adsorption of ^137^Cs and ^90^Sr on radioactively contaminated water samples collected from the watercourses of the Degelen test site at the Semipalatinsk nuclear test site.

The Semipalatinsk Nuclear Test Site, which ranks second in the world and fourth in the number of nuclear weapons tests, was established in 1947 to test Soviet nuclear weapons. From 1949 to 1989, at least 456 nuclear tests were conducted at the Semipalatinsk Nuclear Test site detonating at least 616 nuclear and fusion devices, including 116 atmospheric and 340 underground nuclear blasts. The USSR’s first atomic (1949) and the world’s first hydrogen (1953) bombs were tested here.

Underground nuclear tests were conducted at the Degelen site in horizontal mine workings. From 1961 to 1989, 209 nuclear tests were conducted in 181 adits. A radioecological survey of the Degelen site showed that most of its territory is not contaminated with radionuclides. More than 90% of the radioactivity generated during nuclear tests is concentrated in adit cavities. However, elevated concentrations of radionuclides were detected in natural environments (soil, water, vegetation). They were formed in off-normal situations during nuclear tests; they were accumulated by the environment owing to the opening of adits after the tests and were carried away from the adit cavities by water. The most contaminated areas of the Degelen site are the near-mouth spots of adits with water streams. Long-term monitoring studies show that the carry-over of radionuclides by water from the cavities of nuclear blasts is still ongoing. Altogether, 8 to 12 adits like these have been identified at the Degelen site in different years depending on weather conditions. The peak concentrations of radioactivity in the creek water from different adits were 8.2 × 10^2^ Bq/L for ^137^Cs, 2.1 × 10^3^ Bq/L for ^90^Sr, 6.4 Bq/L for ^239+240^Pu, 2.6 Bq/L for ^241^Am, and 9.9 × 10^5^ Bq/L for ^3^H [44].

The existing radioactively contaminated aquatic ecosystems in the territory of the former Semipalatinsk Nuclear Test Site [45,46,47,48] necessitate the study of technologies for their rehabilitation. This study will provide a scientific basis for the development of a closed-loop technology for the purification of radioactively contaminated natural and wastewater.

## 2. Results and Discussion

### 2.1. Characterisation of Gels

**Figure 1:**
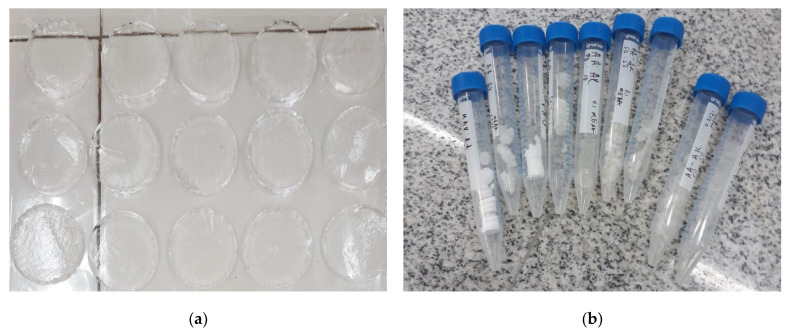
(**a**) Synthesized hydrogels in their swollen form; (**b**) synthesized polymer gels in their dry form.

A series of differently crosslinked hydrogels and cryogels was obtained (Figure 1).

### 2.2. FTIR Spectroscopy Results

**Figure 2:**
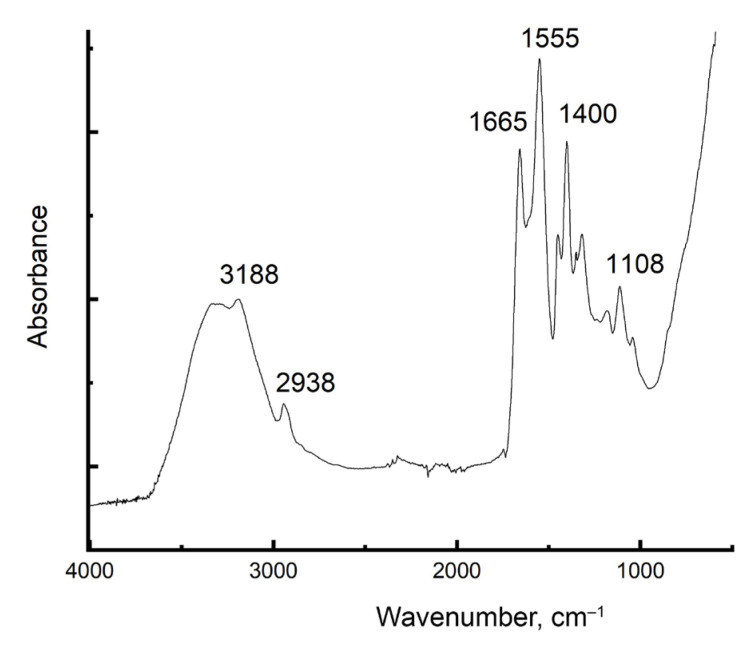
FTIR spectra of hydrogel AM:AA/5.

Figure 2 shows the FTIR spectra of AM:AA/5 hydrogel.

The peak at 3188 cm^−1^ corresponds to N-H stretching from the acrylamide portion of the polymer. The broadness of this peak suggests hydrogen bonding, which is common in amides. The 2938 cm^−1^ peak belongs to C-H stretching (sp^3^ hybridized C-H bonds). The 1665 cm^−1^ peak is characteristic of the C=O stretching (carbonyl group) present in both the acrylamide and acrylic acid units of the polymer. The 1555 cm^−1^ peak is likely due to N-H bending (amide II band) in the acrylamide portion. The 1400 cm^−1^ peak corresponds to C-H bending vibrations in the alkyl chains (–CH_2_–) of the polymer. The 1108 cm^−1^ peak is likely due to C-O stretching in the acrylic acid portion of the polymer.

### 2.3. Swelling Kinetics of Hydrogels

At this point in time, polymer hydrogels based on chemically crosslinked polymers are known to be capable of swelling several dozens or even hundreds of times in aqueous media. The swelling kinetics of gel materials characterizes how many times and over what time the resulted gel will increase in size when immersed in an aqueous solution. Hydrogels were subjected to swelling in an aqueous distilled solution.

**Figure 3:**
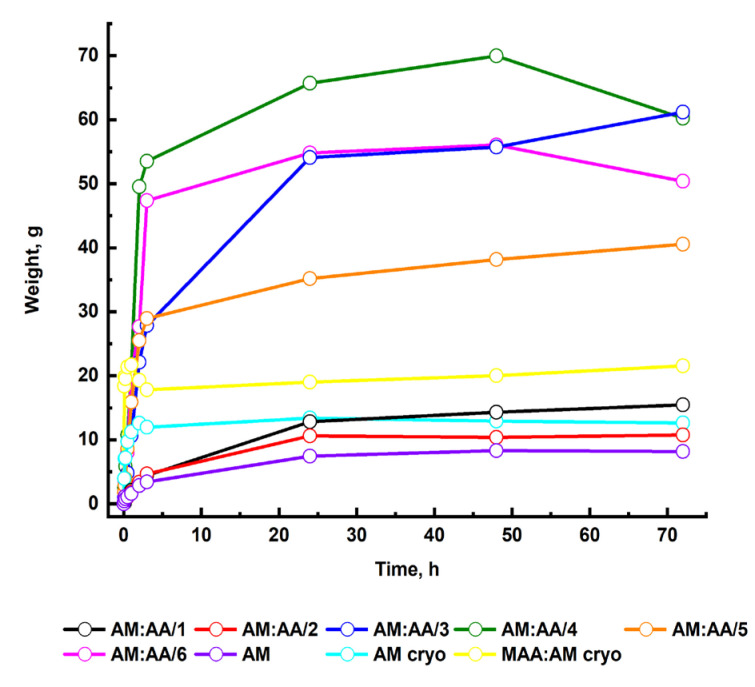
Swelling kinetics of synthesized polymer gels.

A meshy crosslinked structure of a hydrogel provides the penetration of solution’s molecules into a polymer mesh through diffusion, which is accompanied by an increase in the mass and volume of a sample without changing the shape. The graph of mass variation of a polymer gel versus the swelling time is depicted in Figure 3.

On the first day, there is a sharp increase in the degree of swelling of hydrogels and cryogels in all media gradually reaching the equilibrium, which indicates a rapid adsorption of solvent molecules by the hydrogel. The swelling process occurs until the equilibrium of the hydrogel with the aqueous solution is reached, at which the swelling pressure, similar to osmotic, is equilibrated by an elastic strain of the crosslinks.

**Figure 4:**
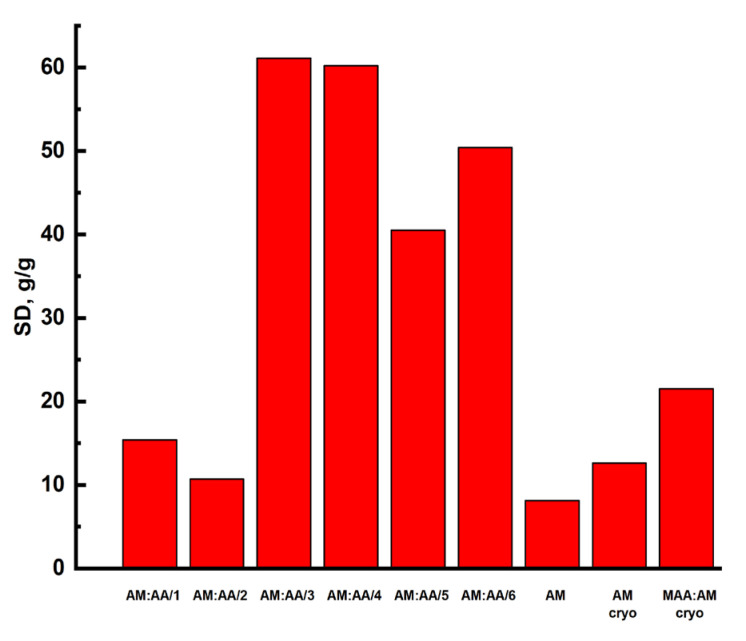
Swelling degree of synthesized polymer gels.

The swelling degree (SD) of synthesized polymer gels is shown in Figure 4.

The highest SD were derived for acrylamide and acrylic acid-based samples; for hydrogel AM:AA/3, this value was 61.1 g/g, for AM:AA/4—60.2 g/g, for AM:AA/6—50.4 g/g, and for AM:AA/5—40.5 g/g. Low values of swelling coefficients for AM (hydrogel) samples—8.1 g/g, AM/2—10.7 g/g, AM (cryogel)—12.6 g/g are associated with a suppressed ionization of carboxyl groups, which leads the samples to a collapsed state.

Since hydrogels can retain water thanks to their three-dimensional polymer mesh, their swelling and swelling rate depend on various parameters such as temperature, pH, ionic intensity, degree of crosslinking, and a type of monomer [49]. A variety of kinetic models of the swelling rate have been presented, but most of them describe either diffusion or polymer relaxation as the predominant process affecting the swelling rate [50]. To understand the mechanism of water adsorption by hydrogels, kinetic data were analyzed using a semi-empirical model proposed by Yavari and Azizyan [51]. This model allows for the description of the swelling process across the SD range and defines whether diffusion or relaxation dominates the process using Equation (1) as follows:

(1) $${SD}_{t\;}={SD}_{e}\;\left[1-e^{\left(-k_{1}t-k_{2}t^{1/2}\right)}\right],$$

where SD_t_—SD of hydrogel (g/g) at a point in time t, SD_e_—equilibrium SD of the hydrogel (g/g), and k_1_ and k_2_—swelling rate constants.

**Figure 5:**
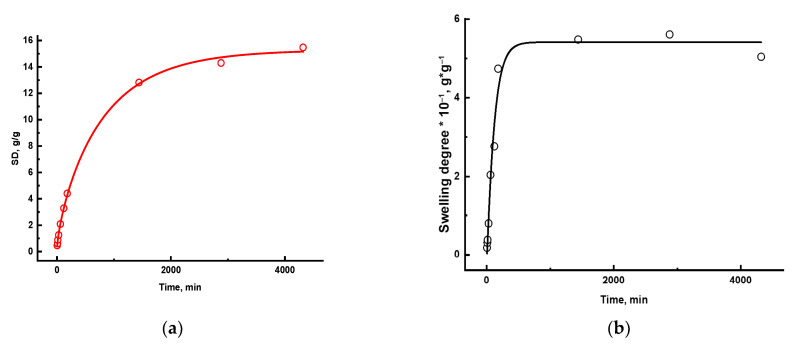
Graphical description of the swelling kinetics of AM:AA/1 hydrogel (**a**); AM:AA/6 hydrogel (**b**) as per the Yavari–Azizyan model.

Figure 5 shows the swelling kinetics of hydrogels synthesized in the distilled water, which was derived from Equation (2).

**Figure 6:**
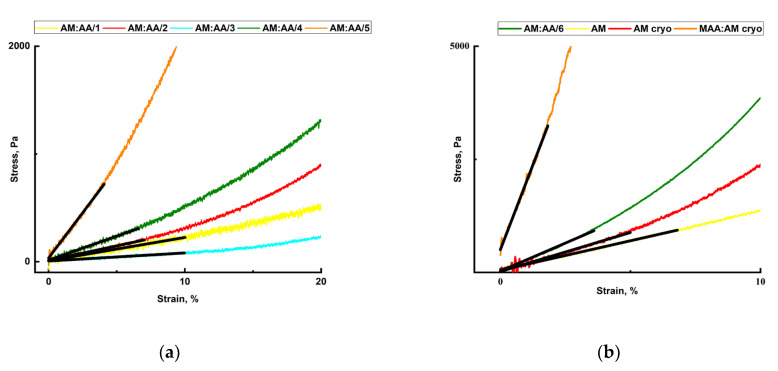
(**a**) Compression graph of synthesized AM:AA/1, AM:AA/2, AM:AA/3, AM:AA/4, and AM:AA/5 hydrogels; (**b**) compression graph of synthesized AM:AA/5, AM, AM (cryo), and MAA:AM (cryo) gels.

The value of the equilibrium degree of swelling is also influenced by the degree of crosslinking of hydrogels and cryogels. The degree of crosslinking is determined by the number of crosslinks in the hydrogel between linear polyacrylic chains and transverse MBAA links. Figure 6 shows that the swelling rate of AM:AA hydrogels was well above AM hydrogels. The findings show a rise in the degree of crosslinking (a decrease in the number of AK monomers between crosslinks) as the concentration of the aqueous AA solution increases, from which AM:AA hydrogels were synthesized. As the degree of crosslinking rises, the values of the equilibrium degrees of sample swelling are observed to decrease, which is associated with more crosslinking and interlacing of monomers that prevent swelling in the structure. A reduction in the degree of crosslinking, in addition to greater stretching of the mesh, often leads to a rupture of the chains, which causes the degree of swelling to rise more.

According to the Yavari–Azizyan model, if k_1_ ≫ k_2_, then the rate of gel swelling is determined by the relaxation of polymer chains. Conversely, if k_1_ ≪ k_2_, the swelling rate is defined by diffusion. If the values of k_1_ and k_2_ are close to each other, both diffusion and relaxation processes affect the swelling rate. Table 1 summarizes the swelling kinetics of the synthesized types of hydrogels.

The data show that for all synthesized hydrogels, the swelling process mechanism corresponds to both diffusion and relaxation processes; however, for AM:AA/1 hydrogel, the swelling rate is defined only by relaxation processes.

### 2.4. Mechanical Properties of Hydrogels and Cryogels

The mechanical characteristics of hydrogels and cryogels, as well as their relationship with the degree of crosslinking, were determined in the regime of uniaxial compression of samples swollen in equilibrium in the distilled water until completely destroyed. Figure 6 shows the compression graphs of synthesized polymer gels.

That said, for all synthesized gels, the elasticity curve looks the same; as the pressure on the sample grows, its elasticity persists to a certain critical point, beyond which the gel begins to break down.

Young’s modulus is a mechanical characteristic of materials that defines their ability to resist longitudinal deformations. It shows the rigidity degree of the material. The approximate value of the modulus can be determined from the strain diagram obtained during compression tests. In this case, Young’s modulus is equal to the ratio of normal strains to the relevant relative deformations in the graph section (Figure 6) up to the limit of proportionality σpc (the tangent of the angle α of the slope of the proportionality section to the deformation axis ε).

E = σ/ε = tgα(2)

**Figure 7:**
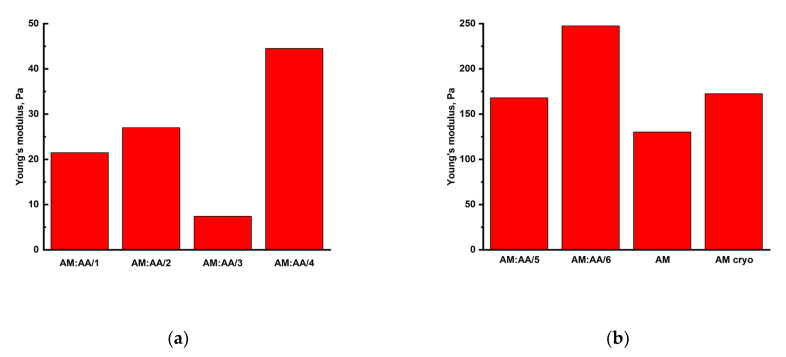
(**a**) Young’s elasticity moduli for each synthesized AM:AA/1, AM:AA/2, AM:AA/3, and AM:AA/4 hydrogel; (**b**) Young’s elasticity moduli for each synthesized AM:AA/5, AM:AA/6, AM, and AM (cryo) gels.

As a result of tests, Young’s elasticity moduli were computed for each synthesized polymer gel as depicted in Figure 7.

As the degree of crosslinking increases, the values of strength, rigidity, and elasticity of the hydrogel samples grow. Due to the greater number of crosslinks, the hydrogel is able to withstand heavy loads without collapsing compared to samples characterized by a lower degree of crosslinking. Similarly, as the degree of gel crosslinking rises, the elasticity modulus of the samples increases. An increase in the elasticity modulus and a decrease in the relative deformation at collapse, as the degree of crosslinking increases, indicate an increase in rigidity and, hence, a slight decrease in the sample elasticity. A rise in the fragility of samples in this case can be explained by a variety of structural flaws, the number of which rises as the degree of crosslinking increases.

### 2.5. TGA Analysis of Hydrogels and Cryogels

**Figure 8:**
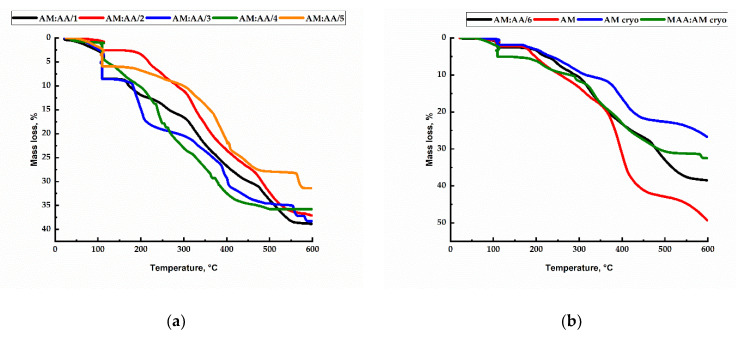
(**a**) Results of the thermogravimetric analysis of each synthesized AM:AA/1, AM:AA/2, AM:AA/3, AM:AA/4, and AM:AA/5 hydrogel; (**b**) results of the thermogravimetric analysis of each synthesized AM:AA/6, AM, AM (cryo), and MAA:AM (cryo) gels.

In order to assess the thermal tolerance, a thermogravimetric analysis of synthesized polymer gels was carried out, which consisted in recording the mass of the test subsample of the substance when its temperature changed. The results of the thermogravimetric analysis are shown in Figure 8.

As can be seen from the graph, the destruction of the resulting gels begins at 100 °C. With a rise in temperature, the gel mass is gradually lost, and at 600 °C polymer gels in each case lose 40 to 50% of their mass.

### 2.6. Study of Cryogel Morphology

**Figure 9:**
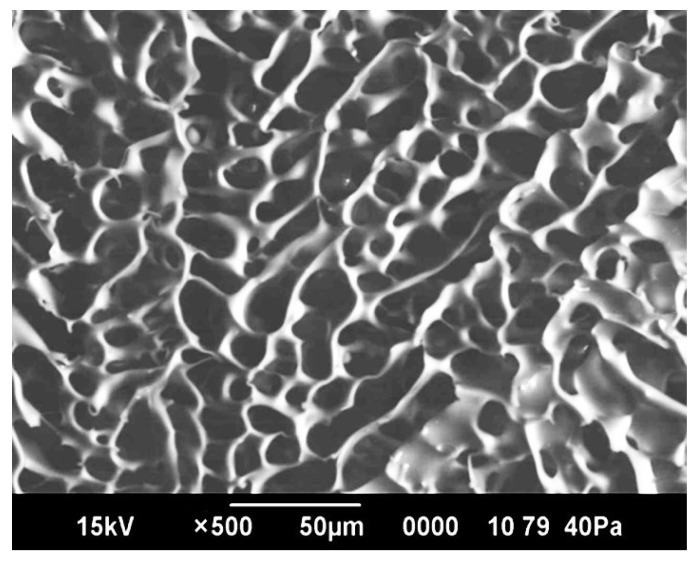
Structure of the synthesized cryogel: an image from the scanning electron microscopy of the cryogel.

Polymer cryogels have a highly porous skeleton structure (Figure 9), which should theoretically facilitate an intensive adsorption of radionuclides into these structures. The pore size in such structures can vary between dozens and hundreds of micrometers. The ImageJ Version 1.54 software was used to calculate the number and pore sizes of synthesized cryogels, which allows for an automated count of cavity sizes based on scanning electron microscopy images.

**Figure 10:**
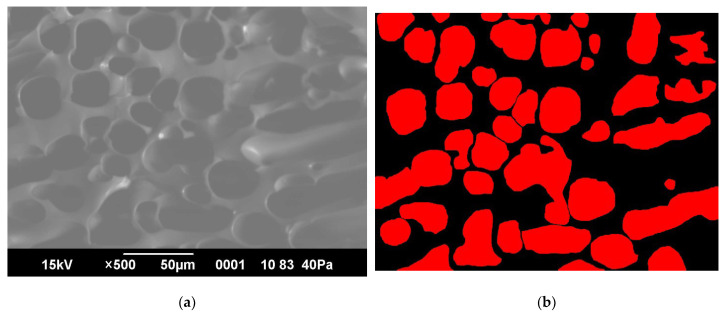
(**a**) Structure of the synthesized cryogel: an image from the scanning electron microscopy of cryogel MAA:AM; (**b**) the counting process of the number and pore size via the application ImageJ.

The figure shows the results of scanning electron microscopy (SEM) and the computational process of the number and pore sizes of synthesized cryogels (Figure 10).

**Figure 11:**
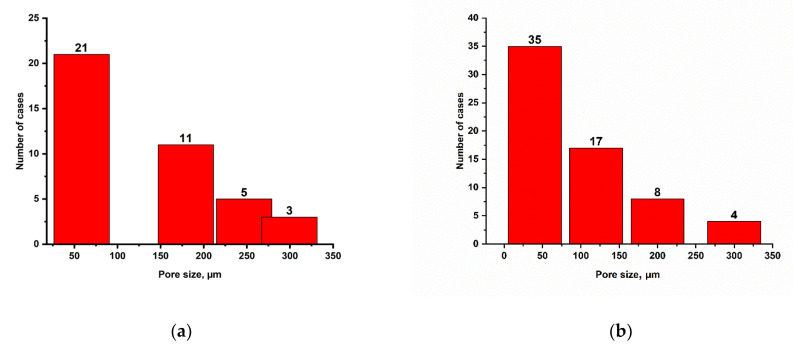
(**a**) Pore size ratio of synthesized AM cryogel to their number; (**b**) pore size ratio of synthesized MAA:AM cryogel to their number.

The figure presents the pore ratio of synthesized cryogels to their number, MAA:AM and AM, via the application ImageJ. The pore size of synthesized cryogel AM averaged from 58 to 350 µm and from 40 to 300 µm for MAA:AM cryogel. The graph shows that most of the pores are smaller than 100 µm. As the pore size increases, their number decreases (Figure 11).

### 2.7. Study of Radionuclide Adsorption

The chemical structure of hydrogels and cryogels enables them to interact with metal ions due to the presence of carboxyl groups from acrylic acid (AA) or methacrylic acid (MAA) in the hydrogel network. The carboxylate groups (–COO^−^) in the hydrogel/cryogel network can participate in ion exchange, where metal ions in the solution replace the hydrogen ions (H^+^) or other cations associated with the carboxylate groups. This is a primary mechanism for metal ion adsorption. On the other hand, the carboxylate and amide groups can act as ligands, forming stable chelate complexes with metal ions. This is particularly effective for transition metal ions, which have vacant d-orbitals available for coordination.

Experiments on the adsorption of ^137^Cs and ^90^Sr from aqueous solutions were conducted under static conditions by placing a polymer gel subsample weighing from 0.07 g to 2.5 g in a 500 mL solution for 72 h. The activity concentration of ^137^Cs in the initial solution ranged from 115 to 250 Bq/L and from 250 to 585 Bq/L for ^90^Sr, respectively.

Table 2 shows the results of gamma-ray spectrometric analyses for the content of ^137^Cs in polymer gel samples following the extraction from a radioactively contaminated aqueous solution.

The tabulated results show that AM:AA/3—2.4 × 10^−8^ mg/g, AM:AA/2—4.1 × 10^−9^ mg/g, and AM:AA/5—3.7 × 10^−9^ mg/g hydrogels have the best adsorption characteristics and selectivity with respect to ^137^Cs. Even though the mass of the first gel increased during adsorption from 0.07 to 12.9 g, that of the second polymer from 2.5 to 66 g, and that of the third adsorbent from 1.2 to 25 g. Polymer cryogels along with other hydrogels proved to be less effective for adsorbing ^137^Cs. Table 3 lists the results of beta spectrometric analyses for the content of ^90^Sr in polymer gel samples after the extraction from a radioactively contaminated aqueous solution.

The tabulated results show that the cryogel MAA:AM—7.0 × 10^−8^ mg/g, hydrogel AM:AA/2, whose activity concentrations was 5.1 × 10^−8^ mg/g, and AM:AA/5—1.5 × 10^−8^ mg/g have the best adsorption characteristics and selectivity with respect to ^90^Sr. Even though the mass of the cryogel increased during adsorption from 0.7 to 10.5 that of the second polymer from 1.2 to 25 g, and that of the third adsorbent from 2.5 to 66.4 g.

**Figure 12:**
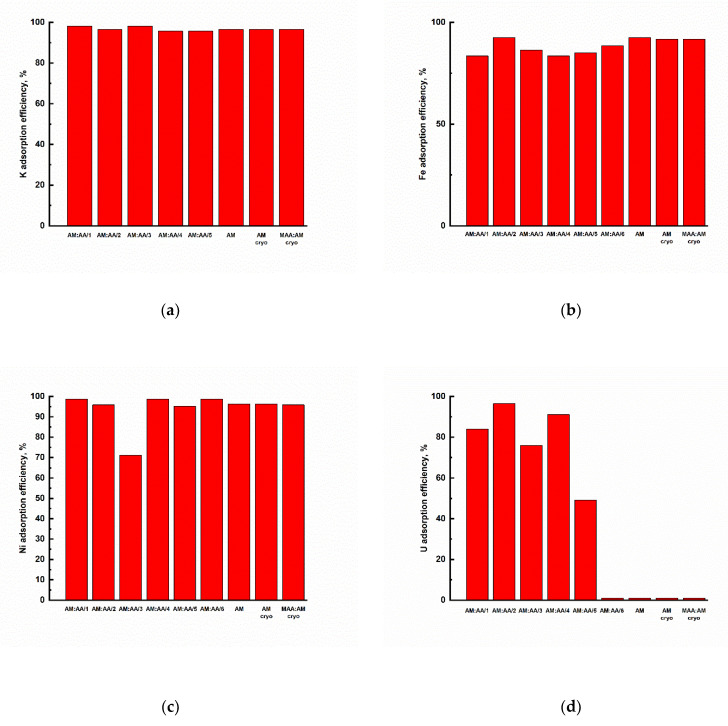
Removal efficiency of synthesized polymer gels relative to: (**a**) K; (**b**) Fe; (**c**) Ni; and (**d**) U.

The adsorption potential of synthesized polymer gels with respect to natural chemical elements K, Fe, Ni, and U unrelated to nuclear tests has also been studied. To do so, the content of the above components was measured in the initial aqueous solutions before adsorption and in the same ones after adsorption. The removal efficiency results are depicted in Figure 12.

The synthesized polymer hydrogels and cryogels demonstrated the best results with respect to the macronutrient K, while in all cases its concentration decreased from 2 to 5 thousand times. Fe content in the aqueous solution decreased hundreds of times after adsorption. The AM:AA/2 and AM hydrogels exhibited the greatest adsorption potential with respect to Fe, while its concentration in the solution decreased from 2 × 10^−1^ mg/L to <1.5 × 10^−2^ mg/L. Ni content in the aqueous solution decreased dozens of time after adsorption. The AM:AA/1 and AM:AA/6 hydrogels demonstrated the greatest adsorption potential with respect to Ni, while its concentration in the solution decreased from 7.7 × 10^−2^ mg/L to <1.5 × 10^−2^ mg/L. For hydrogels AM:AA/1, AM:AA/2, AM:AA/3, AM:AA/4, AM:AA/5, and U content in water samples after adsorption was also reduced from 2 to 11 times. In general, in most cases, the content of all chemical elements tends to drop. However, in some cases, their content after adsorption not only remained the same, but also increased. This increase in the concentration is probably due to the fact that the hydrogel adsorbs water bypassing the element, while increasing the concentration of the element in the solution (Table 4).

The adsorption of metals from aqueous solutions by acrylamide hydrogels and cryogels can occur through several different mechanisms. Acrylamide hydrogels and cryogels, due to their structure, possess certain properties that influence the processes of metal ion adsorption. Acrylamide hydrogels and cryogels contain ions such as hydrogen ions (H^+^) or sodium ions (Na^+^), which can exchange with metal ions in the solution. This process most often occurs in the case of hydrogels and cryogels containing anionic or cationic functional groups, which can bind with metal ions in the solution.

Acrylamide hydrogels and cryogels have polar functional groups, such as amide (–CONH_2_) and carboxyl groups. These groups can interact with metal ions, forming electrostatic bonds. This is especially relevant for hydrogels with anionic functional groups, which can attract cationic metals.

Some functional groups, such as amide (–CONH_2_) or amine groups, can form coordination bonds with metal ions because they have lone pairs of electrons. Such bonds provide stronger adsorption of metal ions, especially for transition metals (e.g., Fe^2^^+^, Ni^2^^+^).

Acrylamide hydrogels and cryogels, like many other hydrogels, have a porous structure. These pores can serve for the physical trapping of metal ions. Ions can enter the pores of the hydrogel and be retained there due to their size or through interactions with functional groups in the pore walls.

In the case of cryogels, which have a more pronounced porous and network structure (such as polyacrylamide cryogels), the capture of metal ions occurs within the microstructure of the material itself. This can be attributed to both physical and chemical interactions, providing high adsorption capacity.

Thus, the adsorption of metal ions by acrylamide hydrogels and cryogels can occur through a combination of several mechanisms, such as ion exchange, electrostatic and coordination interactions, chemisorption, physical adsorption, and pore trapping.

## 3. Conclusions

Over the period of the research work, seven polymer hydrogels and two cryogels based on acrylamide, acrylic acids, and methacrylic acids were synthesized in different proportions and varying in degrees of crosslinking.

The kinetics of synthesized gel materials has been studied. The highest value of the equilibrium degree of swelling of the samples was observed for hydrogels AM:AA/3—61.1 g/g, AM:AA/4—60.2 g/g, AM:AA/6—50.4 g/g, and the lowest ones—for AM (hydrogel)—8.1 g/g, AM:AA/2—10.7 g/g, and AM (cryogel)—12.6 g/g. The data show that for all synthesized hydrogels, the mechanism of the swelling process corresponds to both diffusion and relaxation processes. However, for the AM:AA/1 hydrogel, the swelling rate is only defined by relaxation processes.

The mechanical strength of the synthesized gels was studied. The largest elastic moduli were for cryogels MAA:AM—331.5 Pa and AM—94.09 Pa. The pore size of synthesized cryogel AM averaged from 58 to 350 µm and for MAA:AM cryogel averaged from 40 to 300 µm.

The processes of selective adsorption of radioactive elements in aqueous solutions were investigated. Cryogel MAA:AM—7.0 × 10^−8^ mg/g, hydrogel AM:AA/2—5.1 × 10^−8^ mg/g, and hydrogel AM:AA/5—1.5 × 10^−8^ mg/g have the best adsorption characteristics and selectivity with respect to ^90^Sr. Selectivity with respect to ^137^Cs is well below for hydrogels AM:AA/3—2.4 × 10^−8^ mg/g, AM:AA/2—4.1 × 10^−9^ mg/g, and AM:AA/5—3.7 × 10^−9^ mg/g.

The synthesized polymer hydrogels and cryogels demonstrated the best results with respect to the macronutrient K, for which, in all cases, the concentration decreased from 2 to 5 thousand times. Fe content in the aqueous solution decreased hundreds of times after adsorption. The AM:AA/2 and AM hydrogels demonstrated the greatest adsorption potential with respect to Fe, for which the concentration in the solution was reduced from 2 × 10^−1^ mg/L to <1.5 × 10^−2^ mg/L. Ni content in the aqueous solution dropped dozens of times after adsorption. The AM:AA/1 and AM:AA/6 hydrogels demonstrated the greatest adsorption potential with respect to Ni, for which the concentration in the solution decreased from 7.7 × 10^−2^ mg/L to <1.5 × 10^−2^ mg/L. For hydrogels AM:AA/1, AM:AA/2, AM:AA/3, AM:AA/4, AM:AA/5, and U content in water samples after adsorption also dropped from 2 to 11 times.

## 4. Materials and Methods

### 4.1. Materials

The following reagents were used for the synthesis of polymer hydrogels: acrylamide (AM, purity 99%), acrylic acid (AA, purity 99%), methacrylic acid (MAA, purity 99%), N, N’—methylene bisacrylamide (MBAA, purity 99%) was used as a crosslinking agent. Ammonium persulfate (PSA) 98% purity served as the initiating agent of polymerization. N,N,N′,N′—Tetramethylenediamine (TEMED) used as an initiating agent for cryogel synthesis. The listed reagents were products of Sigma Aldrich (USA) and were used without being extra purified.

### 4.2. Methods

#### 4.2.1. Preparation of Polymer Hydrogels and Cryogels

**Figure 13:**
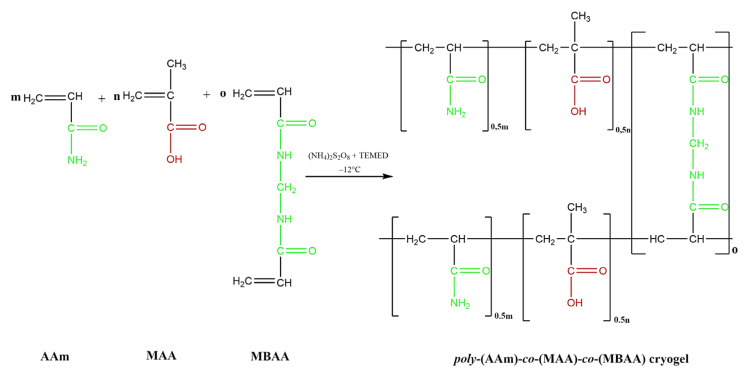
The chemical reaction for hydrogel synthesis.

A mixture of AAm, AA (or MA), and MBAA, corresponding to the molar ratio of AAm:AA (or AAm:MAA) 50:50 (or 10:90) mol%/ mol%, was dissolved in 15 mL of deionized water. Then the required amount of the initiating agent, PSA, was added to the solution and mixed until completely dissolved. A solution containing dissolved monomers, a crosslinking, and initiating agent, was transferred to a test tube and purged with argon for 15 min to remove dissolved oxygen. Thereafter, the test tube was carefully carried to the thermostat. Free-radical copolymerization was accomplished at 60 °C for 20 min. To remove unreacted components and achieve a fully swollen state, all synthesized polymer gels were immersed in distilled water for a week with water daily changed. Next, the prepared hydrogels were sequentially flushed with 25% ethanol (for 2 h), 50% ethanol (for 2 h), 75% ethanol (for 2 h), and 96% ethanol (overnight) for dehydration. For the preparation of dry gels they were then air-dried for 24 h [52,53]. The chemical reaction for hydrogel synthesis is shown in Figure 13.

Polymer cryogels were synthesized using the same procedure as hydrogels, except for the addition of TEMED to the initial solution and creation of free-radical copolymerization conditions. In the case of cryogel synthesis, after adding TEMED, the solution was cooled down in the ice bath for 5 min. The solution in a glass cylinder was frozen, followed by cryopolymerization on a cryothermostat at a temperature of minus 12 °C. To remove unreacted reagents and dry the cryogels, the same procedures were carried out as in the case of hydrogels. The amount of monomers in the initial mixture (mg) used for the synthesis of hydrogels and cryogels is shown in Table 5.

#### 4.2.2. Swelling Kinetics

The swelling degree of hydrogels was calculated as per the formula:

(3) $$\mathrm{SD}=\frac{m_{s}\;{‐\;m}_{d}}{m_{d}},$$

where m_s_—mass of a swollen sample, g.; m_d_—mass of a dried sample, g.

#### 4.2.3. Mechanical Strength of Polymer Gels

The mechanical properties of the hydrogels were examined using a TA.XTplus Texture Analyzer (Stable Micro Systems, Godalming, UK,), as described in a previous study. For this purpose, a hydrogel sample was placed on the platform of the apparatus and compressed using a cylindrical probe (P/75) at a speed of 0.5 mm/s. Young’s moduli of the hydrogels were determined from the slope of the initial linear region of the stress-strain curve for each hydrogel.

#### 4.2.4. Thermogravimetric Analysis of Hydrogels

Thermogravimetric analysis of the freeze-dried hydrogels was performed using a LabSys Evo device (Setaram, Caluire-et-Cuire, France) in the temperature range of 25–600 °C at a heating rate of 10 °C·min^−1^ under an inert atmosphere.

#### 4.2.5. SEM of Cryogels

To analyze the morphology of the cryogels, SEM observations were conducted using a JEOL JSM-6390 LV scanning electron microscope (JEOL, Tokyo, Japan). Sample preparation was performed using a JEE-420 vacuum sputtering unit (JEOL, Tokyo, Japan) with carbon rods (JEOL DATUM, Tokyo, Japan) to form a thin conductive carbon layer on the surface of the cryogels.

#### 4.2.6. Adsorption Experiments

**Figure 14:**
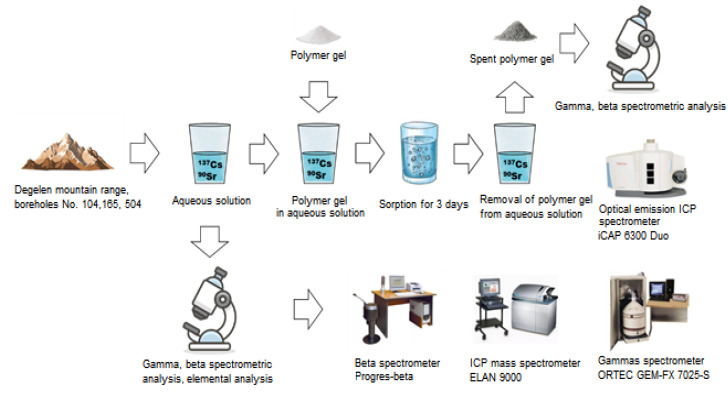
Assessment stages of the selective adsorption efficiency for radioactive elements in aqueous solutions by means of polymer gels.

The efficiency of adsorption of radioactive elements from aqueous solutions by polymer hydrogels and cryogels was evaluated by placing various pure samples of synthesized gels in radioactively contaminated aqueous solutions. The figure shows all the stages of the experiment (Figure 14).

The main point of the experiment was to measure the activity of an aqueous solution before placing a polymer gel there, holding the polymer gel in the aqueous solution for three days, followed by measuring the activity of the polymer gel after removing it from the aqueous solution and evaporation of moisture from the sample.

The main interest in this case was the efficiency of adsorption by polymer gels with respect to such radionuclides as ^90^Sr and ^137^Cs, since, theoretically, hydrogels can effectively adsorb ^90^Sr and ^137^Cs for several reasons. The main factors include their high porosity, large surface for interaction with ^90^Sr and ^137^Cs ions, as well as the ability to chemically bind to ions. Hydrogels have a significant inner surface thanks to their porous structure. This allows them to adsorb and retain ^90^Sr and ^137^Cs ions in their pores. Many hydrogels contain functional groups such as carboxylic, amino groups, or other ions that can react with ^90^Sr and ^137^Cs ions substituting them in the material structure. This occurs due to the ion exchange process, in which ^90^Sr and ^137^Cs ions substitute other ions, such as sodium or calcium in the hydrogel. Hydrogels, especially those based on natural polymers, may contain functional groups that are highly affined to metals, including ^90^Sr and ^137^Cs. For example, groups such as carboxyls or amino groups can effectively bind to ^90^Sr and ^137^Cs ions. Some hydrogels are biodegradable, which makes them suitable for water treatment or the removal of radionuclides such as ^90^Sr and ^137^Cs from the environment. In summary, the combination of chemical properties, porosity, and ion exchange capacity makes hydrogels good candidates for the adsorption of ^90^Sr and ^137^Cs. In addition, these radionuclides are incorporated in the main contaminants of adit water streams at the Degelen site. In addition, the efficiency of adsorption of such natural chemical elements as K, Fe, Ni, and U, which have nothing to do with nuclear tests, has also been studied.

**Figure 15:**
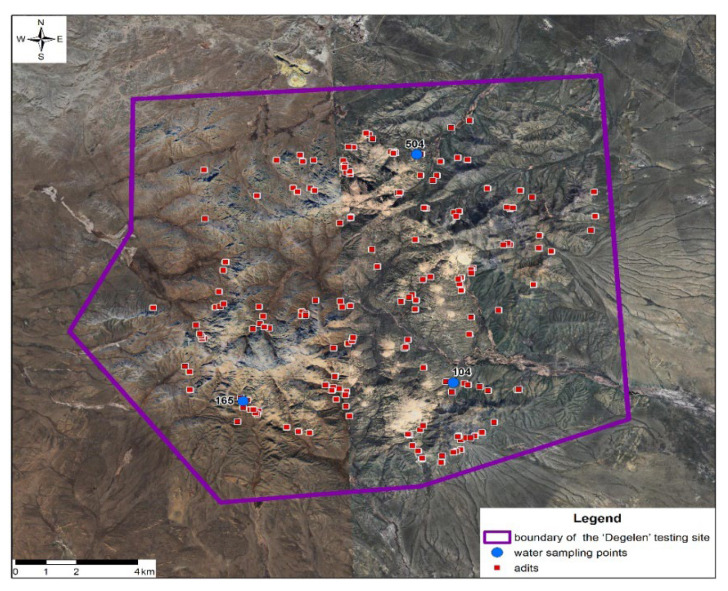
Water sampling points within test adits of the Degelen site.

Water was sampled at the Degelen site in the vicinity of adits No. 504, 165, and 104. Three water samples were collected from each adit totaling 9 L on a 1 L of water per 1 polymer gel basis. Water sampling points are depicted in Figure 15.

The activity of ^137^Cs in the samples of aqueous solutions and polymer gels was measured using an ORTEC GEM-FX 7025-S gamma spectrometer and a Progress-beta beta spectrometer was used to measure the activity of ^90^Sr in the samples. The elemental analysis of water samples was carried out using an ELAN 9000 inductively coupled plasma mass spectrometer and an iCAP 6300 Duo inductively coupled plasma optical emission spectrometer.

## Figures and Tables

**Table 1:** Swelling kinetics of hydrogels as per the Yavari–Azizyan model.

Sample	Se	k_1_	k_2_	R_2_	Primary Process
AM:AA/1	15.28	9.19	0.012	0.99	Relaxation
AM:AA/2	10.62	0.002	0.003	0.99	Relaxation and diffusion
AM:AA/3	5.7	0.004	−0.004	0.99	Relaxation and diffusion
AM:AA/4	6.55	0.011	−0.024	0.98	Relaxation and diffusion
AM:AA /5	3.78	0.009	−0.003	0.99	Relaxation and diffusion
AM:AA /6	5.41	0.009	−0.018	0.98	Relaxation and diffusion
AM	8.19	0.001	0.021	0.99	Relaxation and diffusion

**Table 2:** Adsorption efficiency of synthesized polymer gels relative to ^137^Cs.

Sample Concentration	AA:AM/1	AA:AM/2	AA:AM/3	AA:AM/4	AA:AM/5	AA:AM/6	AM	AM Cryo	MAA:AM Cryo
Aqueous solution activity, Bq/L	260	165	250	260	115	120	165	170	170
Polymer gel activity after adsorption, Bq/g	7.6	13.3	77.3	8.5	12	3.8	2.6	2.9	6.4
Concentration of ^137^Cs in aqueous solution, mg/L	8.1 × 10^−8^	5.1 × 10^−8^	7.8 × 10^−8^	8.1 × 10^−8^	3.6 × 10^−8^	3.7 × 10^−8^	5.1 × 10^−8^	5.2 × 10^−8^	5.3 × 10^−8^
Concentration of ^137^Cs in polymer gel after adsorption, mg/g	2.3 × 10^−9^	4.1 × 10^−9^	2.4 × 10^−8^	2.6 × 10^−9^	3.7 × 10^−9^	1.1 × 10^−9^	8.1 × 10^−10^	9.0 × 10^−10^	1.9 × 10^−9^

**Table 3:** Adsorption efficiency of synthesized polymer gels relative to ^90^Sr.

Sample Concentration	AA:AM/1	AA:AM/2	AA:AM/3	AA:AM/4	AA:AM/5	AA:AM/6	AM	AM Cryo	MAA:AM Cryo
Aqueous solution activity, Bq/L	470	545	448	470	250	277	545	585	585
Polymer gel activity after adsorption, Bq/g	24	260	63	50	80	60	10	35	360
Concentration of ^90^Sr in aqueous solution, mg/L	9.2 × 10^−8^	1.0 × 10^−7^	8.8 × 10^−8^	9.2 × 10^−8^	4.9 × 10^−8^	5.4 × 10^−8^	1.0 × 10^−7^	1.1 × 10^−7^	1.1 × 10^−7^
Concentration of ^90^Sr in polymer gel after adsorption, mg/g	4.7 × 10^−9^	5.1 × 10^−8^	1.2 × 10^−8^	9.8 × 10^−9^	1.5 × 10^−8^	1.1 × 10^−8^	1.9 × 10^−9^	6.9 × 10^−9^	7.0 × 10^−8^

**Table 4:** Adsorption efficiency of synthesized polymer gels relative to K, Fe, Ni, and U.

Gels	Element Concentration in the Adsorbent, mg/g			
	K	Fe	Ni	U
AM:AA/1	2.5 × 10^−3^	0.04 × 10^−3^	0.04 × 10^−3^	0.2 × 10^−3^
AM:AA/2	1.1 × 10^−3^	0.08 × 10^−3^	0.01 × 10^−3^	0.2 × 10^−3^
AM:AA/3	71.4 × 10^−3^	1.3 × 10^−3^	0.7 × 10^−3^	6.2 × 10^−3^
AM:AA/4	0.6 × 10^−3^	0.02 × 10^−3^	0.02 × 10^−3^	0.1 × 10^−3^
AM:AA/5	0.8 × 10^−3^	0.03 × 10^−3^	0.01 × 10^−3^	0.2 × 10^−3^
AM:AA/6	0.001 × 10^−3^	0.05 × 10^−3^	0.03 × 10^−3^	0.001 × 10^−3^
AM	0.6 × 10^−3^	0.04 × 10^−3^	0.01 × 10^−3^	0.001 × 10^−3^
AM cryo	1.2 × 10^−3^	0.08 × 10^−3^	0.01 × 10^−3^	0.02 × 10^−3^
MAA-AM cryo	2 × 10^−3^	0.1 × 10^−3^	0.02 × 10^−3^	0.001 × 10^−3^

**Table 5:** Amount of monomers in the initial mixture that were used to synthesize hydrogels and cryogels.

Polymer Gel	Monomer, g			
	AM	AA	MAA	MBAA
AM:AA/1	0.6	1.2	-	0.075
AM:AA/2	1.1	2	-	0.1
AM:AA/3	0.6	0.6	-	0.075
AM:AA/4	1	1	-	0.1
AM:AA/5	1	1	-	0.2
AM:AA/6	1.8	0.2	-	0.1
AM	2	-	-	0.1
AM cryo	1.2	-	-	0.075
MAA-AM cryo	1.8	-	0.2	0.1

## Data Availability

The data presented in this study are openly available in the article.

## Abbreviations

The following abbreviations are used in this manuscript:

AM	Acrylomide
AA	Acrylic acid
MAA	Methacrylic acid
MBAA	Methylenebisacrylamide
PSA	Ammonium persulfate
TEMED	Tetramethylethylenediamine
SD	Swelling degree
